# Impact of academic center for evidence-based practice star model on door-to-needle times in patients with acute ischemic stroke

**DOI:** 10.12669/pjms.41.3.11056

**Published:** 2025-03

**Authors:** Zhen Li, Lili Zheng, Junke Zheng, Meiping Zhao

**Affiliations:** 1 Zhen Li Nursing Department, Sir Run Run Shaw Hospital, Zhejiang University School of Medicine, Hangzhou, Zhejiang Province 310016, P.R. China; 2 Lili Zheng Emergency Department, Sir Run Run Shaw Hospital, Zhejiang University School of Medicine, Hangzhou, Zhejiang Province 310016, P.R. China; 3 Junke Zheng Emergency Department, Sir Run Run Shaw Hospital Affiliated to Zhejiang University School of Medicine Alar Hospital, Alar, Xinjiang Uygur Autonomous Region 843000, P.R. China; 4 Meiping Zhao Outpatient Department, Sir Run Run Shaw Hospital, Zhejiang University School of Medicine, Hangzhou, Zhejiang Province 310016, P.R. China

**Keywords:** Academic center for evidence-based practice star model, Acute ischemic stroke, Door-to-needle times

## Abstract

**Objective:**

To explore the impact of the academic center for evidence-based practice (ACE) star model on the door-to-needle times (DNT) in patients with acute ischemic stroke (AIS).

**Methods:**

Clinical data of 159 patients with AIS, treated in Sir Run Run Shaw Hospital Afffliated to Zhejiang University School of Medicine Alar Hospital from March 2022 to March 2024, were retrospectively analyzed. Seventy-eight patients received routine care (routine group), and 81 patients were treated using a combination of routine care with the ACE star model (ACE star group). Operating time, intervention effects, activities of daily living (ADL), neurologic outcomes, and incidence of adverse events of the two groups were compared.

**Results:**

The duration of venous opening, computed tomography (CT) examination, and DNT in the ACE star group were shorter than those in the routine group (*P*<0.05). The DNT<45 minutes compliance rate, thrombolytic efficacy, and vascular recanalization in the ACE star group were higher than those in the Routine group (*P*<0.05). After the intervention, the ADL score of the ACE star group was significantly higher than that of the control group, while the NIHSS score was significantly lower than that of the control group (*P*<0.05). There was no significant difference in the incidence of adverse events between the two groups (*P*>0.05).

**Conclusions:**

Adopting routine nursing care and intervention based on the ACE-star model for patients with AIS can shorten DNT, improve thrombolytic effect and vascular recanalization rate. ACE-star model is beneficial for restoring ADL ability and improving neurological function, without significant changes in the occurrence of adverse events.

## INTRODUCTION

Acute ischemic stroke (AIS) is caused by local cerebral tissue circulatory disorders that lead to hypoxia and ischemia, resulting in necrosis, softening of brain tissue, and ultimately, cerebral vascular occlusion.^1^ It was reported that the global prevalence of all stroke subtypes in 2020 was 89.13 million cases, of which the global prevalence of AIS was 68.16 million people.^2^ Moreover, the global stroke incidence in 2020 was 11.71 million, of which ischemic stroke accounted for approximately 65%.^2^ Intravenous thrombolysis is an important modality for the clinical treatment of AIS. It can restore occluded blood vessel reperfusion and cerebral tissue blood perfusion, and rescue ischemic penumbra cells, which is of great significance for improving prognosis.^3^,^4^ Door-to-needle times (DNT) refers to time from hospital admission to the thrombolytic bolus.^5^ Shorter DNT are associated with better thrombolysis effect, reduced degree of neurological damage and improved prognosis.^6^,^7^

In routine nursing, nursing personnel often passively perform relevant operations according to medical orders. This results in low compliance rates for DNT, longer computer tomography (CT) examination time, and increased venous opening time, which greatly affects treatment outcomes of AIS.^8^ Evidence-based nursing can analyze the shortcomings of AIS emergency nursing and develop improvement plans based on evidence-based medicine to enhance the quality and efficiency of nursing services.^9^

The academic center for evidence-based practice (ACE) star model was officially proposed by Dr. Kathleen Stevens at the Academic Center for Evidence-Based Practice located at the University of Texas Health Science Center at San Antonio.^10^ ACE-star is an important evidence-based nursing model in clinical practice, which requires nursing staff to scientifically adopt evidence-based theory to implement nursing interventions by establishing problems, synthesizing evidence, translating into clinical recommendations, integrating practice, and evaluating effectiveness.^10^,^11^ However, there is limited research on the effectiveness of ACE star model in patients with AIS. This retrospective study aimed to clarify the impact of ACE star model on DNT in patients with AIS.

## METHODS

Clinical data of 159 patients with AIS, admitted to Sir Run Run Shaw Hospital Afffliated to Zhejiang University School of Medicine Alar Hospital from March 2022 to March 2024, were retrospectively selected. Patients who received routine nursing interventions were included in the routine group, while patients who received routine nursing combined with interventions based on the ACE star model were included in the ACE star group.

### Ethical approval:

The ethics committee of our hospital approved this study with the number ky2024023, Date: 2024-May-23.

### Inclusion criteria:

- Patients met the AIS diagnostic criteria.^12^
- AIS confirmed through magnetic resonance imaging (MRI) or CT.
- Onset of first stroke.
- Age>18 years old
- The clinical data was complete.

### Exclusion criteria:

- Patients with other organic lesions in the brain.
- Patients with epilepsy.
- Patients with traumatic brain injury.
- Patients with hemorrhagic stroke.
- Patients with kidney, liver, heart and other pathological changes.
- Patients with malignant tumors.
- Patients with contraindications for thrombolysis.

### Routine group:

Routine care was performed as follows: On admission to the emergency room, family members of patients were guided to register. Physicians inquired in detail about the patient’s history and physical examinations they underwent. Laboratory and imaging examination forms were issued, venous channels were established, and patients were accompanied for head CT examinations. After return to the emergency room, doctors reviewed the images and informed patients and family members of thrombolysis-related matters and complications. Patients were given thrombolytic therapy after signing the informed consent form., Vital signs such as blood pressure, respiration, and heart rate were closely monitored during thrombolysis.

### ACE star group:

Intervention in the ACE star group was based on the ACE star model, which employed five key elements - establishment of the problem, synthesis of evidence, translation into clinical recommendations, practice integration, and effect evaluation. Each element was detailed as follows.

Establishment of the problem - intervention team members reviewed and analyzed medical records of patients with AIS received in the past five years, identifying any relevant issues that may potentially affect AIS emergency treatment.

Synthesis of evidence - intervention team members reviewed common issues during historical incidences of nursing care of past cases. Literature searches were performed using Chinese keywords such as “acute ischemic stroke”, “door-to-needle times”, “functional rehabilitation”, “prognosis”, and “nursing intervention”, and English search keywords such as “Acute ischemic stroke”, “door-to-needle times”, “functional rehabilitation”, “diagnosis”, “Nursing intervention”.

Translation into clinical recommendations - Randomized controlled trials were classified as Class A evidence based on the Oxford Center for Evidence-Based Medicine. Non-randomized trial design or randomized design that was not rigorous but the literature have valid evidence was classified as Class B. Case analyses and reviews were classified as Class C. Literature evidence was summarized by intervention team members, and group discussions were organized. Based on past medical records, literature review, and a comprehensive draft of the current AIS emergency nursing process, clinical team standardized and simplified nursing measures related to emergency procedures, including venous opening time, CT examination time, and DNT, in order to improve rescue efficiency. Neurologists and emergency department experts were invited to discuss the shortcomings in the initial draft of the emergency process. Items that not conform to clinical practice were removed, and inappropriate expressions were modified. An emergency nursing process plan for patients with AIS was developed based on ACE star model for continuous quality improvement.

Practice integration - The emergency nursing process plan based on ACE star for continuous quality improvement was implemented. Patient’s vital signs, such as respiration, heart rate, and blood pressure, were checked after receiving emergency treatment. Barthel index and The National Institutes of Health Stroke Scale (NIHSS) were used to assess ADL, and neurological function, respectively. Subsequently, rescue operations were carried out, and one responsible nurse was designated to closely monitor patient’s blood oxygen, blood pressure, and respiration. One nurse established a peripheral venous channel and monitored coagulation function, blood routine, blood biochemistry, and other blood-specific markers. A stroke greenway nurse conducted CT monitoring (CT priority registration examination). Patient’s examination status was systematically evaluated by one neurologist and two emergency physicians. After signing the decision for intravenous thrombolysis treatment, stroke greenway nurses prepared medication according to standardized training, and closely monitored vital signs and medication during the treatment.

Effect evaluation - Intervention team members evaluated the nursing effect, promptly identifying shortcomings of the nursing plan, conducted group discussions and analysis, and summarized the shortcomings. Improvement plans were developed, and nursing quality was continuously improved.

### Collected indicators:

Following indexes were collected from all patients:

- ***Baseline data:*** including gender, age, body mass index (BMI), history of drinking, smoking, diabetes and hypertension.
- ***Operation time for key work steps:*** including venous opening time, CT examination time, and DNT.
- ***Intervention effects:*** including DNT<45 minutes compliance rate, thrombolytic efficacy rate, and vascular recanalization rate. Thrombolysis was considered effective if the NIHSS score decreased by more than four points after the treatment. After vascular recanalization treatment, the degree of vascular recanalization was evaluated using the modified thrombolysis in cerebral infarction (mTICI), and grades 2b and 3 were rated as vascular recanalization.^13^
- ***ADL ability and neurological function:*** ADL ability was evaluated using Barthel index, with a total score of 100 points. Higher score was indicative of better ADL ability.^14^ Neurological function was evaluated based on the NIHSS scale, with a total score of 42 points, and lower score indicating better neurological status. The above evaluation will be conducted before and after intervention.^15^
- The incidence of adverse events, including vascular re-occlusion, intracranial hemorrhage, and gastrointestinal bleeding.

### Statistical Analysis:

Data were analyzed using SPSS version 26.0 (IBM Corp, Armonk, NY, USA). For continuous variables, mean and standard deviation (SD) were calculated. For categorical variables, frequency distribution was provided and expressed as percentage. Chi square tests were used to compare categorical variables between the two groups, such as gender distribution and the presence of diabetes. For continuous variables, independent sample t-test was used for inter group comparison, and paired t-test was used for intra group comparison before and after treatment. P<0.05 was considered statistically significant.

## RESULTS

This study included a total of 159 patients, 82 males and 77 females. Age of patients ranged from 36 to 82 years, with an average of 58.15±10.09 years. Based on the nursing care, 81 patients comprised the ACE star group and 78 patients were included in the Routine group, with no significant difference in baseline data between the two groups (*P*>0.05), Table-I.

**Table-I:** Comparison of baseline data between two groups.

Baseline data	ACE-star group (n=81)	Routine group (n=78)	t/χ^2^	P
Gender (Male/Female)	45/36	37/41	1.049	0.306
Age (Years)	57.41±9.95	58.92±10.25	-0.946	0.236
BMI (kg/m^2^)	23.59±2.72	23.09±2.63	1.191	0.619
History of Drinking (Yes)	43 (53.09)	38 (48.72)	0.303	0.582
Smoking history (Yes)	42 (51.85)	34 (43.59)	1.087	0.297
Diabetes (Yes)	25 (30.86)	32 (41.03)	1.784	0.182
Hypertension (Yes)	43 (53.09)	36 (46.15)	0.764	0.382

***Note:*** ACE = academic center for evidence-based practice; BMI = body mass index.

ACE star method was associated with shorter duration of venous opening, CT examination, and DNT compared to the routine nursing (*P*<0.05), Table-II.

**Table-II:** Comparison of operation time for key links in the related work of two groups (minute)

Group	n	Duration of venous opening	CT examination	DNT
ACE-star group	81	7 (5-8)	19 (16-23)	36 (29-44)
Routine group	78	9 (7-12)	30 (27-36)	44 (36-52)
*Z*		-5.416	-10.285	-4.169
*P*		<0.001	<0.001	<0.001

***Note:*** ACE = academic center for evidence-based practice; CT = computer tomography; DNT = Door-to-needle times.

The DNT<45 minutes compliance rate, thrombolytic efficacy rate, and vascular recanalization rate were significantly higher in the ACE star group compared to the Routine group (*P*<0.05), Table-III. Before the intervention, there was no significant difference in the ADL and NIHSS scores between the two groups (*P*>0.05). After the intervention, ADL scores of the two groups increased, while the NIHSS scores decreased compared to pre-intervention values. Post-intervention ADL score was significantly higher, and NIHSS score was significantly lower in the ACE star group than in the Routine group (*P*<0.05), Table-IV. There was no significant difference in the incidence of adverse events between the two groups (*P*>0.05), Table-V.

**Table-III:** Comparison of intervention effects between two groups.

Group	n	DNT<45 min	Thrombolytic efficiency	Vascular recanalization rate
ACE-star group	81	77 (95.06)	75 (92.59)	53 (65.43)
Routine group	78	67 (85.90)	64 (82.05)	39 (50.00)
*χ^2^*		3.906	4.015	3.881
*P*		0.048	0.045	0.049

***Note:*** ACE = Academic Center for Evidence-based practice; DNT = Door-to-needle times.

**Table-IV:** Comparison of ADL and neurological function between two groups

Time	Group	n	ADL	NIHSS
Before intervention	ACE-star group	81	50.84±6.02	9 (8-11)
	Routine group	78	49.60±6.55	9 (8-10)
	*t/Z*		1.290	-1.676
	*P*		0.199	0.094
After intervention	ACE-star group	81	73.51±5.89^a^	3 (2-4)^a^
	Routine group	78	63.24±5.95^a^	3.5 (3-5)^a^
	*t/Z*		10.927	-3.123
	*P*		<0.001	0.002

***Note:*** Comparison with before treatment in the same group,

a *P*<0.05; ACE = academic center for evidence-based practice; ADL = Activities of Daily Living; NIHSS = National institutes of health stroke scale.

**Table-V:** Comparison of incidence rates of adverse events between two groups.

Group	n	Vascular re-occlusion	Intracranial hemorrhage	Gastrointestinal Bleeding	Overall incidence rate
ACE-star group	81	1 (1.23)	0 (0.00)	1 (1.23)	2 (2.47)
Routine group	78	5 (6.41)	1 (1.28)	2 (2.56)	8 (10.26)
*χ^2^*					2.874
*P*					0.090

***Note:*** ACE = academic center for evidence-based practice

## DISCUSSION

The ACE framework consists of five key elements: knowledge discovery, evidence synthesis or summary, translation into clinical recommendations, implementation into clinical settings, and evaluation.^16^ In the current study, we developed the ACE star model for patients with AIS based on these five key elements. The results of this study showed that in patients with AIS, ACE star method of nursing care is associated with shorter venous opening time, CT examination time, and DNT compared to the routine nursing. The DNT<45 minutes compliance rate, thrombolytic efficacy rate, and vascular recanalization rate were higher in patients who received a combination of routine and ACE star-based nursing compared to patients who received routine nursing alone. ACE star method correlated with lower NIHSS score, and without significant changes in the occurrence of adverse events. Taken together, our results further confirm that the ACE-star model is beneficial in patients with AIS, as it can shorten the time of related key elements of nursing workflow and improve the DNT<45minutes compliance rate. Additionally, ACE star method is able to ensure the effectiveness of thrombolysis and vascular recanalization, and improve neurological function and ADL ability.^17^-^19^

Previous study by Zhong et al.^18^ explored the application value of evidence-based nursing in patients with AIS, and confirmed that evidence-based nursing has a positive significance in improving nursing quality and effectiveness, helping to enhance nursing service quality, improve disease intervention effects, and ensure good outcomes. Gao et al.^19^ showed that nursing services based on evidence-based theory can improve physical symptoms of patients with AIS, alleviate depression and anxiety levels. Moreover, this approach ensures that nursing personnel is proficient in disease intervention skills through a series of evidence-based interventions.

It allows to combine current clinical practices to develop targeted and systematic intervention plans, which can help improve physical and mental status of patients, enhance disease recovery effects, and reduce the risk of complications. Zhong et al.^20^ evaluated the effectiveness of the improved emergency nursing process based on evidence-based theory in patients with AIS, and showed that this intervention model can shorten the time required for DNT and CT examinations, and venous opening, improves ADL ability and cognitive function, reduces the incidence of complications, and increases the success rate of thrombolysis to 94.12%. Our study further supports these conclusions.

Our results demonstrated that ACE star approach in the emergency setting is able to ensure that despite high volumes of patients, accompanying medical staff can communicate effectively with relevant departments in the hospital in a timely manner and hand over patient information. Sufficient preparations to receive patients can save diagnostic time, simplify the process of registration, and allows to start immediate medical treatment,^19^,^20^ ultimately improving the success rate of thrombolysis and the overall effective rate of nursing.^21^,^22^ Liu Guiqin et al.^23^ developed a comprehensive evidence-based nursing plan to treat middle-aged patients with AIS, and showed that it effectively alleviates depression and anxiety in patients, improves their ADL ability, reduces the incidence of complications to 10.0%, and achieves nursing satisfaction of up to 95.0%.

Muñoz et al.^24^ further demonstrated the benefits of evidence-based nursing that employs literature search, evaluates the quality of the retrieved evidence, and establishes intervention plans based on the evidence in combination with the specific conditions of patients. Their results also demonstrated that such evidence-based approach allows to simplify nursing processes, and standardize emergency nursing to provide efficient services for patients. Lee et al.^10^ showed that ACE-star method is an important evidence-based nursing model. Based on the ACE-star theory, nursing interventions can be implemented, and nursing teams can screen for the best evidence of disease care by reviewing medical records and searching relevant literature. This can optimize disease care plans and improve nursing quality.

Cheng et al.^25^ found that ACE Star evidence-based model can intuitively and concisely interpret evidence-based nursing process,^25^ and allow to develop best intervention plans.^10^,^25^ Wang et al.^26^ demonstrated that ACE-Star theory focuses on summarizing and analyzing existing or potential problems before the intervention. Based on the results of the analysis and data review, the nursing process may be divided into five stages, providing patients with systematic nursing services with theoretical support, and continuously improving the intervention plan. Our results further confirm these observations. The ACE Star Model provides a framework for comprehensively and systematically transforming evidence-based practice processes into clinical practice. It highlights the problems encountered, specifies solutions based on evidence-based practices, and considers the knowledge transformation characteristics required in clinical decision-making. This study confirmed that implementing ACE star evidence-based nursing model intervention on patients with AIS on the basis of routine nursing can improve benefits. It also provides reference for the care of patients with AIS during treatment.

### Limitations

This is a single center retrospective study with possible selection bias. In addition, the impact of the two methods on the long-term functional recovery of patients was not analyzed. Future large-scale prospective studies with longer follow-up period are required to validate the results.

## CONCLUSION

In patients with AIS, a combination of routine care and interventions based on the ACE-star model is associated with shorter duration of DNT, and improved thrombolysis and vascular recanalization rate. ACE-star nursing approach is beneficial for restoring ADL ability, and neurological function, without significant changes in the occurrence of adverse events in this group of patients.

### Authors’ Contributions:

**ZL:** Concept, study design, literature search and manuscript writing.

**LZ**, **JZ** and **MZ:** Data collection, data analysis interpretation, critical review.

**ZL:** Revision of manuscript revision and validation. Critical analysis.

All authors have read, approved the final manuscript and are and is responsible for the integrity of the study.
